# The association between APOA1 (rs5069) gene polymorphism and insulin resistance surrogates and metabolic indices among obese individuals with different glycemic statuses (euglycemic and T2DM)

**DOI:** 10.1038/s41598-025-30630-0

**Published:** 2025-12-20

**Authors:** Nagla Usama, Amr E. Ahmed, Salma Mekheimer, Khaled Elhadidy, Mahmoud Farid

**Affiliations:** 1 Medical Laboratory Technology Department, Faculty of Applied Health Science Technology, Misr University for Science and Technology, Cairo, Egypt; 2 Biotechnology and Life Sciences Department, Faculty of Postgraduate Studies for Advanced Sciences, Beni-Suef University, Beni-Suef, Egypt; 3 Internal Medicine Department at Faculty of Medicine, Beni-Suef University, Beni-Suef, Egypt

**Keywords:** Obesity, APOA1 polymorphism, rs5069, Insulin resistance, T2DM, TyG index, Biomarkers, Diseases, Endocrinology, Genetics

## Abstract

Obesity significantly contributes to insulin resistance and type 2 mellitus diabetes (T2DM), with both environmental and genetic factors influencing metabolic risk. Apolipoprotein A1 (APOA1), a key regulator of lipid metabolism, has genetic variants such as rs5069 that may affect metabolic profiles. This study investigated the association between APOA1 (rs5069) polymorphism and metabolic risk among euglycemic and T2DM obese individuals compared to healthy controls. Three hundred participants were enrolled and divided into healthy controls, euglycemic obese, and T2DM obese groups. Demographic, biochemical, and metabolic parameters including fasting blood sugar (FBS), HbA1c, lipid profile, HOMA-IR, TyG index, TyG-BMI, and METS-IR were assessed. APOA1 (rs5069) genotyping was conducted. Statistical analyses included ANOVA, chi-square tests, and principal component analysis (PCA). Obese individuals, particularly those with T2DM, showed significantly elevated insulin resistance markers, dyslipidemia, and metabolic indices (*p* < 0.001) compared to controls. The A allele of APOA1 (rs5069) was more frequent among obese participants. However, no significant differences in metabolic markers were observed among GG, GA, and AA genotypes within either obese group. PCA showed that metabolic variability was driven primarily by insulin resistance and lipid variables rather than genotype. While APOA1 (rs5069) genotype distribution varied across groups, it did not independently impact metabolic risk. Insulin resistance and dyslipidemia are the main contributors to metabolic disturbances in obesity, supporting the utility of non-invasive markers for early risk assessment.

## Introduction

Obesity has emerged as a significant global health concern, markedly heightening the risks of insulin resistance, dyslipidemia, and type 2 diabetes mellitus (T2DM), which subsequently increase the prevalence of cardiovascular illnesses and other obesity-related comorbidities^1^. Although environmental influences are significant, genetic predisposition is essential in influencing individual susceptibility to metabolic diseases^2,3^.

The APOA1 gene, encoding apolipoprotein A-I, plays a crucial role in lipid metabolism as the primary protein of high-density lipoprotein (HDL). Its function in reverse cholesterol transport (RCT), lipid binding, and activation of lecithin cholesterol acyltransferase (LCAT) is essential for maintaining lipid homeostasis^4^. The APOA1 (rs5069) polymorphism, characterized by a cytosine (C) to thymine (T) transition at + 83 bp in the first intron, has been linked to alterations in HDL function and cholesterol efflux^5^. Although this polymorphism is in a non-coding region, it may influence gene expression by affecting regulatory elements such as enhancer activity or splicing mechanisms. Thus, rs5069 is considered a potentially functional polymorphism that could modulate APOA1 expression and subsequently impact lipid metabolism and associated metabolic conditions^6^. This polymorphism was selected for analysis due to evidence suggesting its potential role in metabolic disorders. Studies have reported that genetic variations in APOA1 are associated with altered serum lipid levels, obesity, and insulin resistance. Specifically, the rs5069 variant has been implicated in modulating HDL-C concentrations, influencing cardiovascular risk factors, and potentially affecting metabolic syndrome components. Numerous studies have shown that ApoA1 has anti-obesity effects, and alterations in body fat mass were observed in mice when circulating ApoA1 levels were altered. Mice given ApoA1 mimetics, for instance, lost weight quickly and had less adipose tissue mass overall^7^.

Moreover, non-invasive surrogate indicators of insulin resistance, such the triglyceride-glucose (TyG) index, TyG-BMI, and Metabolic Score for Insulin Resistance (METS-IR), have become increasingly favored as effective instruments for evaluating metabolic health beyond conventional metrics^8^. Surrogate insulin resistance indices serve as more straightforward and practical alternatives to insulin-based markers of insulin resistance for clinical application^9^. However, their interaction with genetic variants such as APOA1 rs5069 in the context of obesity and diabetes is not well established. And the interaction between APOA1 genetic variants and these non-insulin-based insulin resistance surrogates remains largely unexplored. Importantly, data on the APOA1 (rs5069) polymorphism among Egyptian populations are limited, despite the high regional prevalence of obesity and T2DM and possible ethnic differences in allele frequency and metabolic response. Therefore, this study was designed to assess metabolic and biochemical differences among euglycemic obese and T2DM obese individuals compared to healthy controls, and to investigate the potential role of APOA1 (rs5069) polymorphism in modulating these metabolic traits. We further employed principal component analysis (PCA) to explore multidimensional relationships between genotype and metabolic indices.

## Materials and methods

A total of 300 Egyptian adults aged 35–55 years were recruited and classified into three groups: 100 euglycemic obese individuals (31% males, 69% females; BMI ≥ 30), Individuals with BMI ≥ 30 kg/m^2^ and a confirmed diagnosis of type 2 diabetes mellitus based on the American Diabetes Association (ADA) criteria (fasting blood glucose ≥ 126 mg/dL and/or HbA1c ≥ 6.5%), with a known disease duration of more than 5 years, and 100 healthy controls (29% males, 71% females; BMI < 30).

The sample size (n = 300) was determined based on feasibility and availability of eligible participants during the recruitment period, while ensuring sufficient statistical power for detecting moderate effect sizes in genetic and biochemical analyses. The sample size (n = 300; 3 × 100) was primarily convenience-based but provides acceptable statistical power for the main planned analyses: it is powered to detect small–medium effects in one-way ANOVA (Cohen’s f ≈ 0.18–0.20) and medium effects in pairwise comparisons (Cohen’s d ≈ 0.5). For allele frequency comparisons the study is powered to detect moderate to large differences in MAF (absolute differences ~ 0.12–0.15).

Participants were recruited from the diabetic clinic at Souad Kafafi University Hospital over a defined period (e.g., January 2023 to December 2023). Eligible individuals were approached during their routine clinic visits and provided with detailed information about the study’s objectives, procedures, and potential risks and benefits. Those who expressed interest underwent a screening process to assess eligibility based on predefined inclusion and exclusion criteria. Each case provided written informed consent, and the MUST University Ethics Committee (FWA00025577) approved the research protocol.

Inclusion Criteria: Adults aged 35–55 years of both sexes, classified according to BMI and glycemic status. Diagnosis of T2DM was confirmed based on ADA Standards of Care in Diabetes—2025 guidelines^10^.

Exclusion Criteria: Participants with a history of chronic liver or kidney disease, autoimmune or infectious diseases, pregnancy, breastfeeding, smoking, or use of medications affecting glucose or lipid metabolism were excluded.

Data Collection and Biochemical Assessment: Demographic variables including age, sex, and BMI were recorded. Biochemical analyses included fasting blood sugar (FBS), HbA1c, fasting insulin, and lipid profile parameters. Blood samples were collected after an overnight fast using the following tubes: EDTA tubes for DNA extraction and HbA1c measurement, Sodium fluoride tubes for FBS, Plain tubes for lipid profile and fasting insulin. FBS and lipid profiles were measured using an XL180 fully automated clinical chemistry analyzer. LDL-C was calculated using the Friedewald equation. HbA1c was measured via the turbidimetric inhibition immunoassay, and insulin levels were assessed using the Chemux Bioscience ELISA kit (Cat. No. 1080).

### Metabolic and insulin surrogate indices

#### Glycation Hb index (HGI)

Baseline FBS and HbA1c data were used to estimate the linear relationship between FBS and HbA1c in the study population. Predicted HbA1c was calculated by inserting the corresponding FBS value into the linear regression equation (HbA1c = 0.01313 FBS (mg/dL) + 6.17514). HGI was calculated by subtracting the predicted HbA1c from the observed HbA1c^11^.

HOMA-IR index was calculated to evaluate insulin resistance^12^.

NHHR was computed as non-HDL-C [mg/dl]/HDL-C [mg/dl]. TC [mg/dl] − HDL-C [mg/dl] equals non-HDL-C [mg/dl]^13^.

The TG/HDL-C ratio was calculated by dividing the serum concentration of TG by HDL-C measured in mg/dL^14^.

The IR surrogate indicators were calculated using the following formula^15^.

$${\text{TyG index }}\left( {\text{Triglyceride Glucose Index}} \right) \, = {\text{ log }}\left( {{\text{fasting TG}} \times {\text{FPG}}/{2}} \right)$$

$${\text{TyG}} - {\text{BMI }}\left( {{\text{Triglyceride glucose}} - {\text{body mass index}}} \right) \, = {\text{ TyG }} \times {\text{ BMI}}$$

$${\text{METS}} - {\text{IR}} = {\text{Ln }}\left( {{2} \times {\text{fasting glucose }}\left[ {{\text{mg}}/{\text{dL}}} \right] + {\text{fasting triglyceride }}\left[ {{\text{mg}}/{\text{dL}}} \right]} \right) \times {\text{BMI}}/{\text{Ln }}\left( {{\text{fasting HDL}} - {\text{C }}\left[ {{\text{mg}}/{\text{dL}}} \right]} \right)$$

For genetic analysis, the study utilized the Thermo Scientific Gene JET Whole Blood Genomic DNA Purification Mini Kit for DNA extraction. The APOA1 gene polymorphisms (rs 5069) was performed using the Applied Biosystems TaqMan SNP Genotyping Assays, Location: Chr.11:116837538 on Build GRCh38 Context Sequence [VIC/FAM] GAAGACCTCAGGTACCCAGAGGCCC[G/A] GCCTGGGGCAAGGCCTGAACCTTGA, which employ TaqMan 5nuclease chemistry to amplify and detect specific polymorphisms in purified genomic DNA samples. The PCR process was conducted in a 10 μl reaction mixture, including TaqMan Master Mix, assay working stock, and DNA sample, following a standardized cycling protocol: initial denaturation at 95 °C, followed by 40 cycles of denaturation and annealing/extension, with a final extension at 60 °C. This methodology ensured accurate detection and analysis of the targeted genetic polymorphisms and biochemical markers in the study population. Genotyping quality control procedures were performed to ensure data accuracy. Approximately 5% of randomly selected DNA samples were re-genotyped to verify reproducibility, yielding 100% concordance.

#### Statistical analysis

Data was analyzed using SPSS version 26. Quantitative variables were summarized as mean ± standard deviation (SD), and categorical variables as frequencies and percentages. The normality of data distribution was assessed using the Shapiro–Wilk test and visual inspection of histograms. ANOVA was used for group comparisons, with Duncan’s post hoc test applied for multiple comparisons. Genotype frequencies were tested for Hardy–Weinberg equilibrium using the chi-square test. A *p*-value < 0.05 was considered statistically significant. PCA of biochemical and metabolic parameters stratified by APOA1 genotypes was conducted using R (version 4.5) and RStudio (version 2025.05.1 + 513).

## Results

Table 1 displays the demographic and clinical characteristics of the studied groups:

**Table 1:** the compare means values (µ ± SD) for demographic, biochemical and metabolic indices among three studied groups using ANOVA and Duncan test.

Parameters	Control N = 100	Euglycemic obese N = 100	T2DM obese N = 100	*P*-value
Sex				*X*^*2*^ = 1.830 *P*-value = 0.401
M =	29	31	38	
F =	71	69	62	
Age	42.7 ± 3.76^a^	43.6 ± 5.28^a^	42.85 ± 5.48^a^	0.384
BMI	24.64 ± 2.2^a^	35.63 ± 5.03^b^	32.88 ± 3.42^c^	< 0.001
Fasting Insulin µIU/mL	5.32 ± 2.76^a^	6.07 ± 4.12^a^	8.30 ± 3.6079^b^	< 0.001
FBS mg/dl	85.59 ± 9.0^a^	91.91 ± 11.16^a^	132.33 ± 40.6^b^	< 0.001
HbA1C %	4.735 ± 0.41^a^	4.96 ± 0.50^b^	7.87 ± 1.19^c^	< 0.001
TC mg/dl	151.7 ± 27.25^c^	182.6 ± 37.90^b^	181.2 ± 40.16^b^	< 0.001
TG mg/dl	125.9 ± 30.87^a^	167.23 ± 55.20^b^	148.78 ± 51.01^c^	< 0.001
VLDL mg/dl	25.19 ± 6.17^a^	33.44 ± 11.04^b^	29.75 ± 10.20^c^	< 0.001
HDL-C mg/dl	55.06 ± 9.04^a^	53.75 ± 8.18^b^	50.04 ± 9.0^b^	< 0.001
LDL-C mg/dl	71.44 ± 27.15^a^	95.40 ± 35.04^b^	101.4 ± 37.65^b^	< 0.001
non-HDL/HDL	150.7 ± 27.25^a^	181.6 ± 37.90^b^	180.2 ± 40.16^b^	< 0.001
TG/HDL	2.376 ± 0.79^a^	3.18 ± 1.15^b^	3.12 ± 1.37^b^	< 0.001
Glycation Hb index	2.64 ± 0.41^a^	2.41 ± 0.50^b^	-0.49 ± 1.19^c^	< 0.001
HOMA.IR	1.12 ± 0.59^a^	1.37 ± 0.92^a^	2.71 ± 1.50^b^	< 0.001
TyG Index	8.5 ± 0.29^a^	8.88 ± 0.35^b^	9.08 ± 0.60^c^	< 0.001
TyG-BMI	210.64 ± 19.58^a^	316.9 ± 47.5^b^	298.94 ± 38.96^c^	< 0.001
METS-IR	35.17 ± 3.7^a^	52.47 ± 7.41^b^	50.83 ± 7.39^b^	< 0.001

Data are expressed as mean ± SD. Superscript letters (a, b, c) denote significant differences between groups according to Duncan’s post hoc test (*p* < 0.05).

BMI, body mass index; FBS, fasting blood sugar; HbA1c, glycated hemoglobin; TC, total cholesterol; TG, triglycerides; VLDL, very low-density lipoprotein; HDL-C, high-density lipoprotein cholesterol; LDL-C, low-density lipoprotein cholesterol; HOMA-IR, homeostasis model assessment for insulin resistance; TyG, triglyceride–glucose index; METS-IR, metabolic score for insulin resistance.

The study population comprised 300 participants, who were evenly distributed into three groups: healthy controls (n = 100), euglycemic obese individuals (n = 100), and obese individuals with type 2 diabetes mellitus (T2DM) (n = 100). Statistical analysis revealed no significant differences in sex distribution among the groups (χ^2^ = 1.830, *p* = 0.401). Similarly, there were no significant differences in mean age across the groups (*p* = 0.384), indicating that the study groups were demographically comparable and well-matched.

A comprehensive comparison of metabolic and biochemical parameters among the three groups was conducted using one-way ANOVA followed by Duncan’s post hoc test. The analysis revealed significant differences across nearly all variables as follows:

Body mass index (BMI) was markedly elevated in both obese groups compared to the control group (*p* < 0.001), with the highest values recorded among euglycemic obese individuals. Measures of insulin resistance, including fasting insulin and homeostasis model assessment of HOMA-IR, were significantly higher in the T2DM obese group than in both the euglycemic obese and control groups (*p* < 0.001).

The glycemic indicators, FBS was significantly higher in the T2DM obese group compared to both control and euglycemic obese groups, while the difference between euglycemic obese and controls was not statistically significant. In contrast, HbA1c levels increased progressively from controls to euglycemic obese to T2DM obese individuals, with all pairwise differences reaching statistical significance.

Lipid profile parameters also showed significant differences. Total cholesterol (TC), triglycerides (TG), very low-density lipoprotein (VLDL), and low-density lipoprotein cholesterol (LDL-C), as well as derived ratios such as non-HDL/HDL and TG/HDL, were significantly elevated in both obese groups compared to controls (*p* < 0.001). In contrast, high-density lipoprotein cholesterol (HDL-C) was significantly lower in both obese groups compared to controls (*p* < 0.001).

Additionally, the glycation hemoglobin index was significantly reduced in the T2DM group compared to both euglycemic obese and control groups (*p* < 0.001). Furthermore, surrogate markers of insulin resistance, including the (TyG) index, TyG-BMI, and METS-IR, were significantly higher in the obese groups compared to controls (*p* < 0.001). These results support the clinical utility of these indices in identifying individuals at elevated metabolic risk.

### APOA1 (rs5069) genotype and allele distribution

The Hardy–Weinberg equilibrium (HWE) test was applied to compare observed versus expected genotype frequencies for the APOA1 rs5069 single nucleotide polymorphism (SNP) across the four study groups. The results indicated statistically significant differences in the distribution of rs5069 genotypes among the groups.

The frequency distribution of APOA1 (rs5069) genotypes and alleles across study groups is presented in Table 2. The GG genotype was most prevalent in the control group (65%), but its frequency was significantly lower among euglycemic obese (40%) and T2DM obese (48%) individuals. In contrast, the GA genotype increased in frequency in the obese groups, while the AA genotype was absent in the control group but present in 11% and 15% of the euglycemic and T2DM obese groups, respectively. These differences were statistically significant (*p* < 0.001).

**Table 2:** The frequency distribution APOA1 (rs 5069) genotypes in all studied cases.

APOA1 genotyping & alleles. rs5069	Control group N = 100	euglycemic obese N = 100	T2DM obese N = 100	*P*- value		
				Control Vs euglycemic obese	Control Vs T2DM obese	euglycemic obese Vs T2DM obese
GG (ref)	65 (65%)	40 (40%)	48 (48%)	< 0.001	0.011	< 0.001
GA	35 (35%)	49 (49%)	37 (37%)			
AA	0	11 (11%)	15 (15%)			
G (ref)	165 (82.5%)	129 (64.5%)	133 (66.5%)	< 0.001	0.005	0.674
A	35 (17.5%)	71 (35.5%)	67 (33.5%)			

Allelic analysis showed a significantly higher frequency of the A allele in both euglycemic obese (35.5%) and T2DM obese (33.5%) individuals compared to controls (17.5%) (*p* < 0.001), suggesting a possible association between the A allele and obesity-related phenotypes.

### Metabolic profiles across APOA1 genotypes

Within each obesity subgroup, one-way ANOVA was used to compare biochemical and metabolic indices across APOA1 genotypes.

**Fig. 1:**
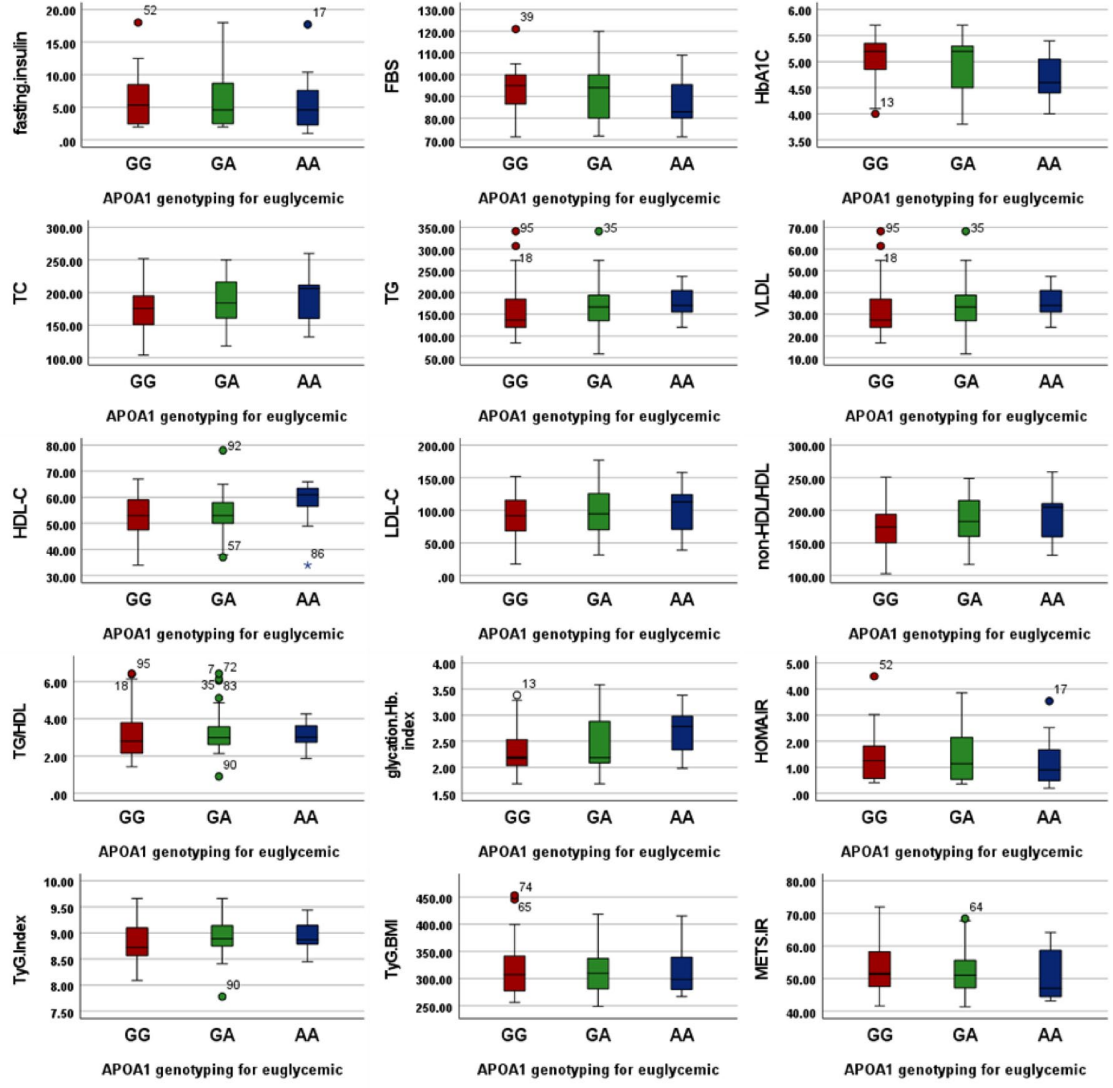
Boxplots illustrate the distribution of biochemical and metabolic indices among different APOA1 (rs5069) genotypes (GG, GA, and AA) in euglycemic obese individuals. Each box represents the interquartile range (IQR) with the horizontal line indicating the median; whiskers denote the minimum and maximum values within 1.5 × IQR. Red boxes = GG genotype. Green boxes = GA genotype. Dark Blue boxes = AA genotype. Outliers are displayed as individual points. Units of measurement: Fasting insulin (µIU/mL), FBS (mg/dL), HbA1c (%), TC, TG, VLDL, HDL-C, LDL-C, non-HDL/HDL (mg/dL), Glycation Hb index (unitless), HOMA-IR (unitless), TG/HDL (ratio), TyG index (unitless), TyG-BMI (index), METS-IR (score).

Among euglycemic obese individuals (Table 3 and Fig. 1), no statistically significant differences were found in any measured parameters across GG, GA, and AA genotypes (*all p-values* > *0.05*). Although numerical variations existed, particularly in lipid profiles and insulin resistance indices, these did not reach statistical significance.

**Table 3:** the compare means values (µ ± SD) for biochemical and metabolic indices among APOA1 (rs 5069) genotypes for euglycemic obese individuals using ANOVA.

Parameters	GG	GA	AA	*P*-value
Fasting Insulin µIU/mL	6.0 ± 3.7	6.2 ± 4.3	5.8 ± 5.0	0.95
FBS mg/dl	93.5 ± 10.2	91.6 ± 11.9	87.8 ± 11.3	0.31
HbA1C %	5.0 ± 0.5	5.0 ± 0.5	4.7 ± 0.5	0.10
TC mg/dl	175.4 ± 36.7	186.3 ± 38.0	192.2 ± 40.8	0.27
TG mg/dl	157.0 ± 60.1	173.4 ± 53.5	177.3 ± 40.7	0.31
VLDL mg/dl	31.4 ± 12.0	34.7 ± 10.7	35.5 ± 8.1	0.31
HDL-C mg/dl	52.9 ± 8.4	53.5 ± 7.6	57.9 ± 9.3	0.19
LDL-C mg/dl	91.1 ± 33.6	98.1 ± 36.0	98.8 ± 37.7	0.61
non-HDL/HDL	174.4 ± 36.7	185.3 ± 38.0	191.2 ± 40.8	0.27
TG/HDL	3.1 ± 1.3	3.3 ± 1.1	3.1 ± 0.7	0.62
Glycation Hb index	2.3 ± 0.5	2.4 ± 0.5	2.7 ± 0.5	0.10
HOMA.IR	1.4 ± 0.9	1.4 ± 0.9	1.3 ± 1.0	0.90
TyG Index	8.8 ± 0.4	8.9 ± 0.4	8.9 ± 0.3	0.46
TyG-BMI	318.1 ± 49.4	316.1 ± 45.7	316.4 ± 53.4	0.98
METS-IR	53.0 ± 7.4	52.3 ± 7.4	51.3 ± 8.3	0.79

**Fig. 2:**
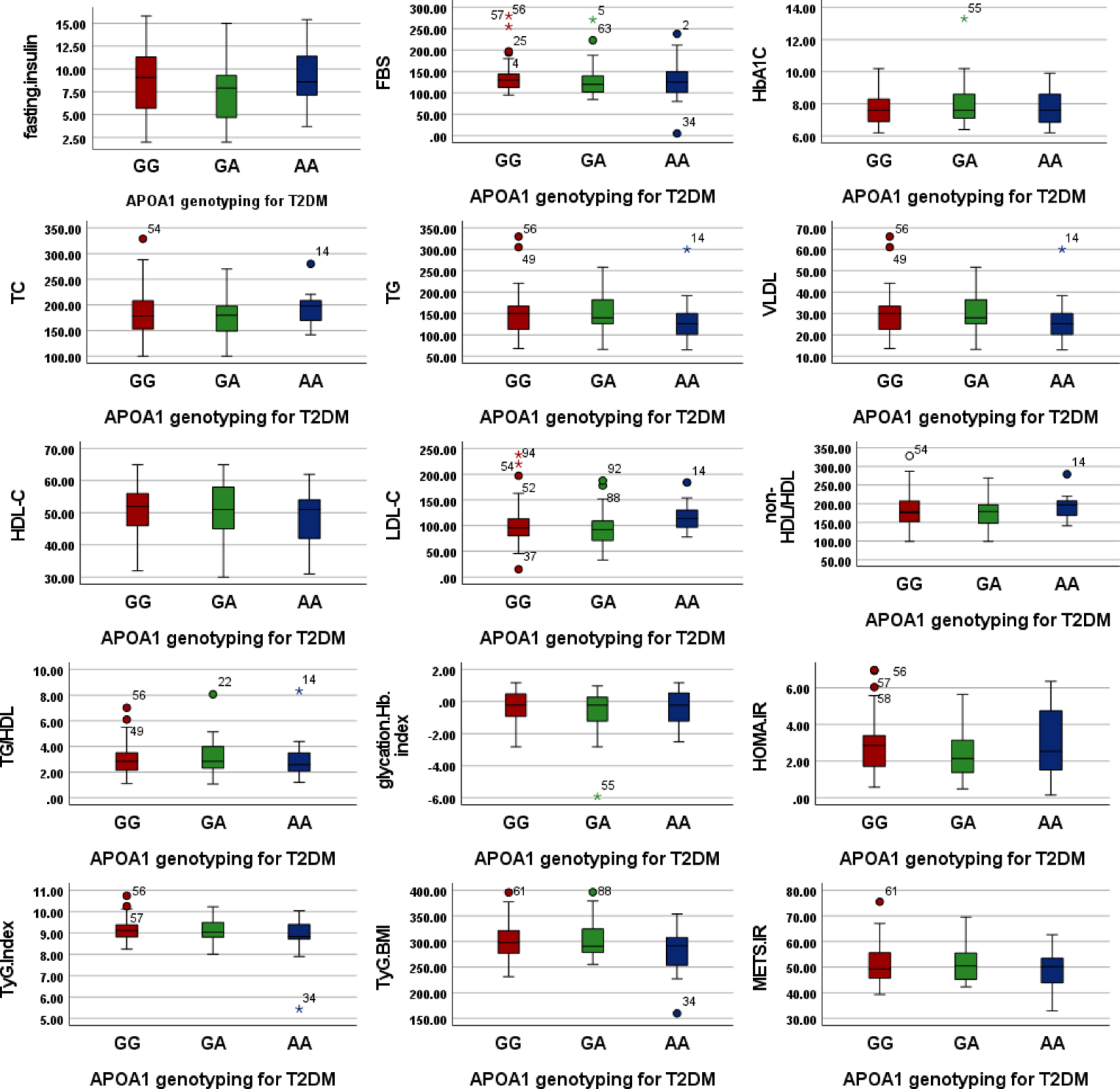
Boxplots showing the distribution of biochemical and metabolic indices among APOA1 (rs5069) genotypes (GG, GA, and AA) in T2DM obese individuals. Color coding and units are identical to those in Fig. 1.

Similarly, in T2DM obese individuals (Table 4 and Fig. 2), there were no significant differences in metabolic parameters across genotypes. A trend toward lower TyG-BMI in the AA genotype was observed but was not statistically significant (*p* = 0.082).

**Table 4:** the compare means values (µ ± SD) for biochemical and metabolic indices among APOA1 (rs 5069) genotypes for T2DM obese individuals using ANOVA.

Parameters	GG	GA	AA	*P*-value
Fasting Insulin µIU/mL	8.5 ± 3.8	7.6 ± 3.3	9.4 ± 3.6	0.21
FBS mg/dl	136.9 ± 37.1	128.6 ± 39.1	127.1 ± 54.6	0.56
HbA1C %	7.8 ± 1.1	8.0 ± 1.3	7.8 ± 1.2	0.83
TC mg/dl	182.7 ± 43.5	174.6 ± 37.6	192.8 ± 33.9	0.31
TG mg/dl	148.3 ± 52.5	154.6 ± 47.6	136.0 ± 55.4	0.49
VLDL mg/dl	29.7 ± 10.5	30.9 ± 9.5	27.2 ± 11.1	0.49
HDL-C mg/dl	50.7 ± 8.8	49.9 ± 9.5	48.1 ± 9.5	0.61
LDL-C mg/dl	102.3 ± 40.7	93.7 ± 35.2	117.5 ± 28.8	0.11
non-HDL/HDL	181.7 ± 43.5	173.6 ± 37.6	191.8 ± 33.9	0.31
TG/HDL	3.1 ± 1.3	3.2 ± 1.3	3.0 ± 1.7	0.80
Glycation Hb index	− 0.5 ± 1.1	− 0.6 ± 1.3	− 0.4 ± 1.2	0.83
HOMA.IR	2.9 ± 1.5	2.4 ± 1.3	3.0 ± 1.9	0.2
TyG Index	9.1 ± 0.5	9.1 ± 0.5	8.8 ± 1.1	0.16
TyG-BMI	303.0 ± 37.3	302.0 ± 35.0	278.3 ± 48.7	0.08
METS-IR	51.3 ± 7.6	51.2 ± 7.0	48.5 ± 7.8	0.41

### PCA analysis of metabolic and biochemical profiles stratified by APOA1 genotypes

To further explore the relationship between APOA1 (rs5069) genotypes and metabolic phenotypes, principal component analysis was performed separately for euglycemic obese and T2DM obese groups, with metabolic and biochemical parameters projected as vectors and individuals stratified by genotypes.

**Fig. 3:**
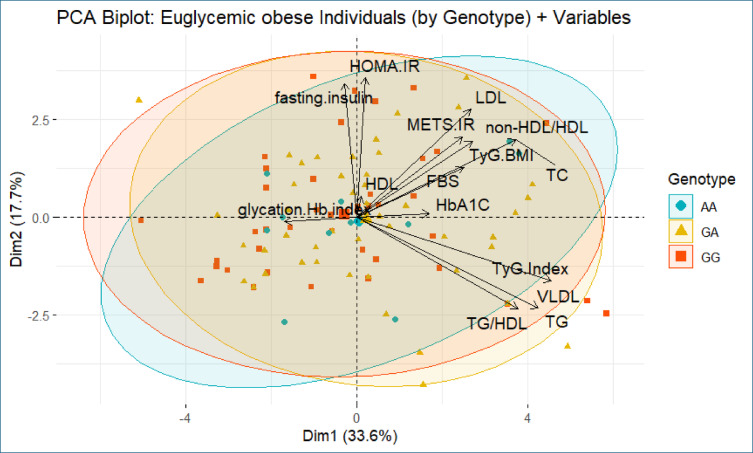
Principal Component Analysis (PCA) biplot of metabolic and biochemical parameters among euglycemic obese individuals stratified by APOA1 (rs5069) genotypes. The plot displays individuals carrying the GG (red squares), GA (yellow triangles), and AA (blue circles) genotypes. The first two principal components (Dim 1 and Dim 2) explain 33.6% and 17.7% of the total variance, respectively. Vectors represent the direction and magnitude of contribution of metabolic variables, including lipid markers, insulin resistance indices, and glycemic measures. Despite some variation, the genotypes show overlapping distribution, with no clear separation.

In the euglycemic obese group (Fig. 3), the first two principal components (PC1 and PC2) accounted for 51.3% of the total variance (33.6% and 17.7%, respectively). The PCA biplot revealed overlapping distributions among individuals carrying the GG, GA, and AA genotypes, with no distinct separation. Key contributors to the variance included lipid-related variables such as TG, TG/HDL ratio, VLDL, TyG index, and TyG-BMI which loaded strongly on the first principal component. Insulin resistance markers, including fasting insulin and HOMA-IR, contributed primarily to the second dimension. Notably, HDL-C and glycation hemoglobin index were inversely oriented relative to most metabolic risk indicators, suggesting a protective effect.

**Fig. 4:**
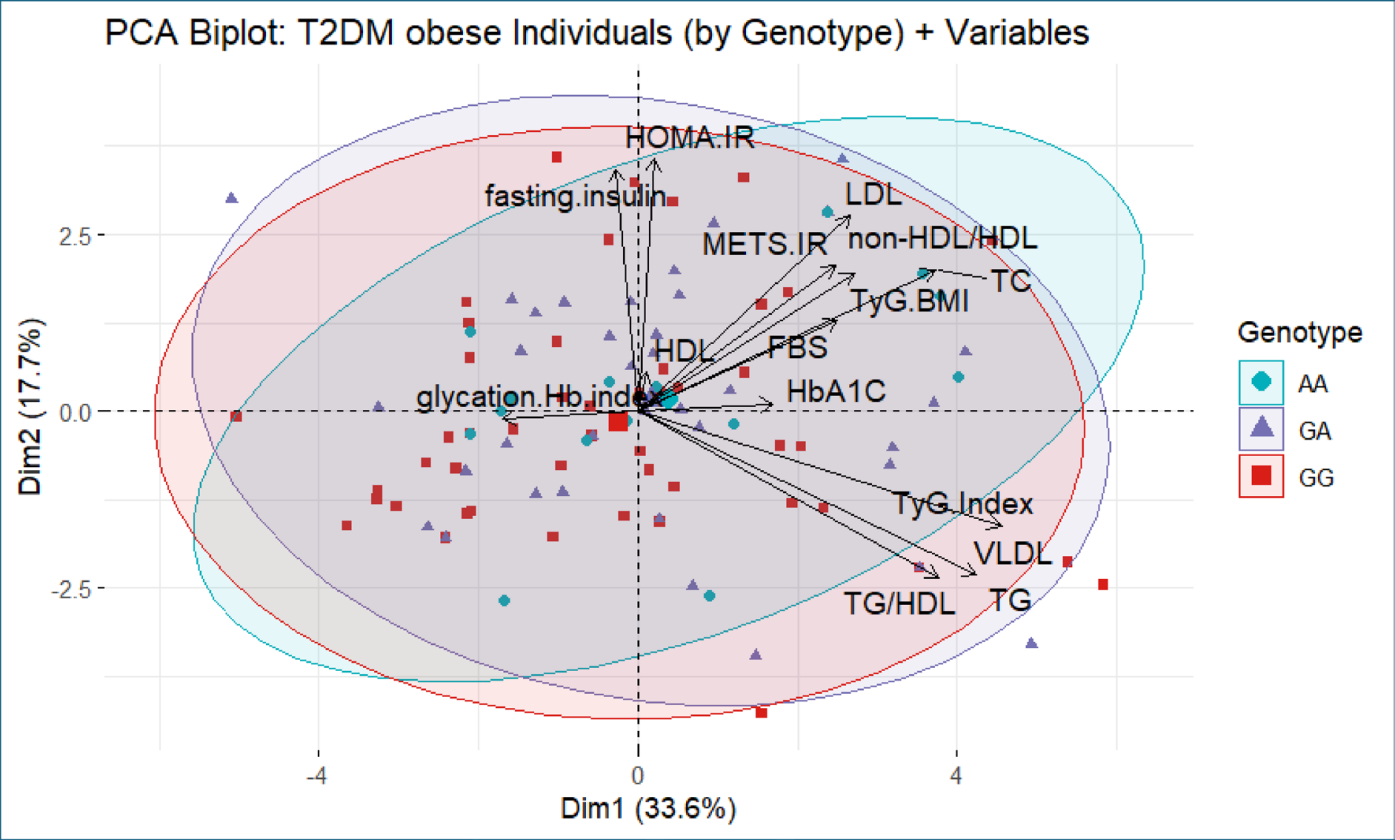
Principal Component Analysis (PCA) biplot of metabolic and biochemical parameters among T2DM obese individuals stratified by APOA1 (rs5069) genotypes. Colored points represent genotypes: GG (red squares), GA (purple triangles), and AA (blue circles). Dim 1 and Dim 2 account for 33.6% and 17.7% of the total variance, respectively. lipid-related indices (e.g., TG, TyG, TyG-BMI) and insulin resistance markers (e.g., HOMA-IR, fasting insulin) are major contributors to variance. No distinct clustering of individuals by genotype was observed.

Similarly, in the T2DM obese group (Fig. 4), PC1 and PC2 explained the same proportion of total variance. The distribution of APOA1 genotypes again showed substantial overlap, with no discernible clustering pattern. As in the euglycemic group, the dominant contributors to variance were lipid indices and insulin resistance markers. Although individuals with the AA genotype exhibited slight clustering in the left region of the biplot, this did not correspond to a distinct metabolic profile.

Overall, PCA results indicate that metabolic variability in both euglycemic and T2DM obese individuals is primarily driven by biochemical and insulin resistance markers, rather than APOA1 genotype. These findings are consistent with the ANOVA results, which demonstrated no statistically significant differences in metabolic parameters across genotypes within each group.

## Discussion

This study investigated the metabolic and biochemical profiles of obese individuals with differing glycemic statuses and explored the potential association of APOA1 (rs5069) gene polymorphism with metabolic risk. The findings underscore the profound impact of obesity and glycemic status on insulin resistance and lipid abnormalities while also suggesting that APOA1 genotype may not independently influence these metabolic outcomes within obese subgroups.

As expected, both euglycemic and T2DM obese individuals exhibited significantly higher BMI values compared to control group, consistent with previous reports linking excess adiposity to adverse metabolic effects^16^. Our findings further revealed marked elevations in fasting insulin, HOMA-IR, and surrogate markers such as the TyG index, TyG-BMI, and METS-IR, particularly among the T2DM obese group. These elevations reflect progressive deterioration in insulin sensitivity with advancing metabolic dysfunction. These results align with prior research demonstrating the utility of these non-invasive indices in capturing early metabolic risk and insulin resistance in obese populations^17–22^.

The study also revealed significant alterations in lipid profiles among obese individuals, especially those with T2DM. Elevations in total cholesterol, triglycerides, VLDL, and LDL-C, along with reductions in HDL-C, support the well-established role of obesity and insulin resistance in promoting atherogenic dyslipidemia^23–27^. The TG/HDL and non-HDL/HDL ratios, which are often used as predictors of cardiovascular risk, were also significantly elevated in the obese groups. These findings underscore the need for comprehensive lipid monitoring in individuals with obesity, particularly those at risk of or diagnosed with T2DM^28–30^.

Interestingly, while APOA1 (rs5069) genotype and allele distributions differed significantly across the study groups, with the A allele being more frequent among obese individuals, this variation did not translate into significant differences in metabolic or biochemical parameters within each subgroup. Neither traditional lipid markers nor insulin resistance indices differed significantly among GG, GA, and AA genotypes in either the euglycemic or T2DM obese groups. These findings suggest that although APOA1 polymorphism may be associated with obesity predisposition, it does not appear to independently modulate the severity of metabolic disturbance once obesity is established^31–34^.

Previous studies conducted in diverse populations have explored the metabolic relevance of the APOA1 rs5069 polymorphism, with somewhat inconsistent findings across ethnic groups. In European cohorts, the A allele of rs5069 has been linked to reduced serum apolipoprotein A-I (ApoA1) and high-density lipoprotein cholesterol (HDL-C) concentrations, supporting its potential role in modulating lipid metabolism and cardiovascular risk^35^. ApoA1, the principal structural protein of HDL particles, is critical for reverse cholesterol transport; therefore, variants that diminish their expression or function could predispose to atherogenic dyslipidemia.

In contrast, studies from Asian populations have reported variable effects. Shioji et al.^36^ observed that certain APOA1 polymorphisms, including rs5069, were associated with higher HDL-C and lower triglyceride levels among Japanese individuals, suggesting a potentially protective lipid profile. Conversely, Wang et al.^37^ demonstrated that the APOA1 rs5069 variant interacted with obesity to increase the risk of low HDL-C disease in the Xinjiang pastoral population of China, highlighting the role of gene–environment and gene–obesity interactions in modulating metabolic outcomes. Moreover, Balcıoğlu et al.^38^ found that rs5069 was associated with increased coronary artery disease (CAD) risk independent of traditional lipid measures in East Asian populations, suggesting the presence of alternative biological pathways influencing cardiovascular susceptibility beyond classical lipid metabolism.

These ethnic disparities in the metabolic impact of APOA1 rs5069 may reflect differences in allele frequencies and linkage disequilibrium patterns. According to global genomic databases, the minor allele frequency (MAF) of rs5069 varies markedly among populations—being relatively higher in Asian and European ancestries compared to African cohorts—which may partly explain inconsistent genotype–phenotype associations across studies^39^. Collectively, these findings emphasize the importance of population-specific investigations, such as the present study in an Egyptian cohort, to clarify the contribution of APOA1 genetic variation to metabolic risk and to determine whether its effects are influenced by ethnicity, obesity status, or environmental exposures.

This interpretation is further supported by principal component analysis (PCA), which showed that variability in metabolic parameters was primarily driven by insulin resistance and lipid markers rather than genotype. The absence of clear clustering by genotype reinforces the notion that the APOA1 variant does not contribute significantly to the heterogeneity in metabolic risk among obese individuals. This aligns with previous studies suggesting that metabolic dysfunction in obesity results from a complex interplay of genetic, environmental, and behavioral factors^40,41^.

The strengths of this study include the comprehensive assessment of conventional and emerging metabolic indices, the incorporation of genetic analysis, and the use of PCA to explore multidimensional patterns in the data. However, some limitations must be acknowledged. The cross-sectional design restricts the ability to infer causality between APOA1 rs5069 genotype, insulin resistance, and metabolic disturbances. Although the sample size was sufficient to detect group-level differences, it may have been underpowered to identify small genotype-specific effects. Furthermore, only one genetic variant (APOA1 rs5069) was examined; other potentially relevant genetic markers, epigenetic factors, and gene–environment interactions were not assessed.

Lifestyle and environmental factors such as diet, physical activity, and socioeconomic status were also not measured, which may have influenced the observed metabolic differences. These factors are known to play a major role in lipid metabolism and insulin sensitivity, and accumulating evidence suggests that APOA1 variants interact with dietary composition to modulate lipid profiles. For instance, individuals carrying APOA1 risk alleles have shown differential responses in HDL-C and triglyceride levels depending on dietary fat intake and overall diet quality^42,43^. Similarly, gene–diet interactions involving APOA1 polymorphisms have been reported in Asian and European populations, where nutrient patterns, physical activity, and lifestyle behaviors significantly affect lipid and glucose metabolism^36^ Future research should therefore integrate detailed dietary assessments and lifestyle characterization to better understand how APOA1 rs5069 influences metabolic risk within diverse.

In conclusion, this study demonstrates that insulin resistance and dyslipidemia are major determinants of metabolic disturbances among obese individuals, independent of glycemic status or APOA1 rs5069 genotype. Although the APOA1 variant may predispose to obesity, it does not appear to independently affect metabolic risk once obesity is established. Notably, the TyG index and METS-IR emerged as practical, low-cost, and non-invasive markers for early identification of metabolic risk. These indices may be particularly useful in resource-limited settings where genetic testing is unavailable. Future large-scale, longitudinal studies are needed to validate these findings and explore the interplay between APOA1 polymorphisms, lifestyle factors, and metabolic health across diverse populations.

## Data Availability

The analysis in this study used the human reference genome assembly GRCh38, available from NCBI under accession number GCA_000001405.
